# Effect of low blood pressure on prognosis of acute heart failure

**DOI:** 10.1038/s41598-024-66219-2

**Published:** 2024-07-06

**Authors:** Hyun-Jin Kim, Sang-Ho Jo

**Affiliations:** 1 Division of Cardiology, Department of Internal Medicine, Hanyang University College of Medicine, Hanyang University Guri Hospital, Guri, Korea; 2 Cardiovascular Center, Hallym University Sacred Heart Hospital, Anyang-si, Korea; 3 Division of Cardiology, Department of Internal Medicine, Hallym University College of Medicine, Hallym University Sacred Heart Hospital, 22, Gwanpyeong-ro 170beon-gil, Dongan-gu, Anyang-si, Gyeonggi-do Republic of Korea

**Keywords:** Cardiovascular biology, Cardiology, Diseases, Health care, Medical research, Risk factors

## Abstract

Low blood pressure (BP) is associated with poor outcomes in patients with heart failure (HF). We investigated the influence of initial BP on the prognosis of HF patients at admission, and prescribing patterns of HF medications, such as angiotensin-converting enzyme inhibitors (ACEi), angiotensin receptor blockers (ARB), and beta-blockers (BB). Data were sourced from a multicentre cohort of patients admitted for acute HF. Patients were grouped into heart failure reduced ejection fraction (HFrEF) and HF mildly reduced/preserved ejection fraction (HFmrEF/HFpEF) groups. Initial systolic and diastolic BPs were categorized into specific ranges. Among 2778 patients, those with HFrEF were prescribed ACEi, ARB, or BB at discharge, regardless of their initial BP. However, medication use in HFmrEF/HFpEF patients tended to decrease as BP decreased. Lower initial BP in HFrEF patients correlated with an increased incidence of all-cause death and composite clinical events, including HF readmission or all-cause death. However, no significant differences in clinical outcomes were observed in HFmrEF/HFpEF patients according to BP. Initial systolic (< 120 mmHg) and diastolic (< 80 mmHg) BPs were independently associated with a 1.81-fold (odds ratio [OR] 1.81, 95% confidence interval [CI] 1.349–2.417, p < 0.001) and 2.24-fold (OR 2.24, 95% CI 1.645–3.053, p < 0.001) increased risk of long-term mortality in HFrEF patients, respectively. In conclusion, low initial BP in HFrEF patients correlated with adverse clinical outcomes, and BP < 120/80 mmHg independently increased mortality. However, this relationship was not observed in HFmrEF/HFpEF patients.

## Introduction

Heart failure (HF) is a global health concern of immense significance owing to its escalating prevalence and high mortality rate^1,2^. Central to the pathophysiology of HF is the regulation of blood pressure (BP) which plays a crucial role in managing this condition^3^. Elevated BP levels can lead to left ventricular hypertrophy and subsequent HF, whereas a lower BP can indicate an advanced stage of the disease and serve as a prognosticator of poor outcomes^4^. However, the intricate relationship between BP and HF, particularly the implications of admission BP across different HF subtypes during acute HF episodes, remains relatively underexplored^2^. Several studies have sought to understand the association between BP at admission and long-term clinical outcomes in patients with acute HF^5^. However, these studies often overlooked the considerable variation among HF subtypes, which may conceal critical prognostic details. For example, lower BP levels typically characterize the HF subtype with reduced ejection fraction (HFrEF), which often suggests a severe disease state and unfavourable prognosis^6^. On the other hand, HF with preserved ejection fraction (HFpEF) usually presents with higher BP levels, but the prognosis related to this pattern remains unclear^7^. In addition, the identification of optimal systolic and diastolic BP thresholds upon admission that are capable of predicting all-cause mortality in HF patients, particularly those with HFrEF, is of utmost importance^8^. Such thresholds could have a significant impact on risk stratification, guideline tailored treatment strategies, and improve survival rates^2,9^.

This study aimed to explore the association between BP at hospital admission and subsequent long-term clinical outcomes in acute HF patients. Specifically, we outlined this relationship across two distinct HF subsets: HFrEF and heart failure with mildly reduced or preserved ejection fraction (HFmrEF/HFpEF). Recognizing the complexities of HF management, the study also sought to identify initial BP thresholds upon admission that might correlate with poorer clinical outcomes. It is crucial to emphasize that these initial measurements aid in early risk stratification and must be integrated with ongoing clinical assessments and medication adjustments to manage HF over time comprehensively.

## Methods

### Study design and setting

Our data were collected from the KorHF registry, a comprehensive multicentre cohort study that included patients hospitalized with acute HF in South Korea^10,11^. The study period was from June 2004 to April 2009. The cohort consisted of 3200 patients across 24 different hospitals in Korea, all of whom were diagnosed with acute HF upon admission using the Framingham criteria. The diagnosis was confirmed at the time of discharge based on clinical assessment and patient response to initial treatment. It was strongly recommended that all patients undergo at least 1 year of follow-up. Information on outcomes, such as rehospitalization owing to HF and death, was gathered from both medical records and telephone interviews, and these data were prospectively recorded. Of the 3200 acute HF patients originally recruited, admission BP data were available for 3112. Within this subset, echocardiographic data on left ventricular ejection fraction (LVEF), a key measure of left ventricular (LV) systolic function, were accessible for 2778 patients. The 2778 patients with available LVEF data were further categorized based on their HF classification and initial BP. Of these, 1594 patients were classified as having HFrEF because their LVEF was < 40%. The remaining 1184 patients with LVEF ≥ 40% were categorized as having HFmrEF/HFpEF. Additionally, initial systolic blood pressure (SBP) was subdivided into five categories: < 100, 100–119, 120–139, 140–159, and ≥ 160 mmHg. The diastolic blood pressure (DBP) was likewise classified into < 50, 50–69, 70–89, 90–109, and ≥ 110 mmHg.

The study adhered to the principles outlined in the Declaration of Helsinki and was approved by the Institutional Review Board of Hallym University Sacred Heart Hospital (IRB no. 2002-S2005) as well as by each participating hospital. Prior to inclusion in the study, all the patients provided written informed consent.

### Data collection

Using a web-based electronic data capture system, we compiled patient demographic and clinical details from electronic case report forms in the KorHF registry database. We analysed these data to extract the baseline characteristics and conventional cardiovascular risk factors. Key laboratory results pertinent to the prognosis of HF were also obtained. LV systolic function was assessed by calculating LVEF. LVEF was gauged using a modified Simpson’s biplane method in apical four- and two-chamber views. If this method was not applicable, the M-mode technique was employed for LVEF measurements. Moreover, we acquired echocardiographic parameters for the LV end-diastolic and end-systolic dimensions. Upon discharge, we meticulously logged the array of prescribed medications. This encompasses angiotensin-converting enzyme inhibitors (ACEi), angiotensin receptor blockers (ARB), and beta-blockers (BB), all of which are critical to the patient's BP management post-discharge.

### Study outcomes

The primary outcome assessed was the occurrence of all-cause mortality, determined through an evaluation of medical records or via telephonic conversations with family during the follow-up period (average 472.5 ± 416.6 days, median 362.5 days). Additionally, the incidence of combined events, such as all-cause mortality or readmission for HF, was recorded. Readmission for HF was defined as hospitalization due to the deterioration of HF symptoms.

### Statistical analyses

Categorical data were presented as frequencies and percentages, and means and standard deviations were employed for continuous variables. Pearson’s Chi-square test was used to compare categorical variables, whereas the Student’s *t*-test was used to compare normally distributed continuous variables. For non-normally distributed continuous variables, the Mann–Whitney *U* test was used. A linear-by-linear test was used to derive the trends of study outcomes and baseline categorical data relative to the BP categories. The ideal cut-off points for SBP and DBP that could potentially predict all-cause mortality in patients with HFrEF were explored using receiver operating characteristic (ROC) curve analyses. Kaplan–Meier survival analyses and log-rank tests were used to compare death-free survival rates between the groups stratified by the initial SBP and DBP cut-off values. Univariate and multivariate Cox proportional hazards regression analyses were conducted to identify predictors of all-cause mortality in the HFrEF group after adjusting for individual risk factors. Any variables that exhibited predictive significance (p-value < 0.05) during the univariate analysis were then fed into the regression model. The threshold for significance was set at p < 0.05. All analyses were performed using SPSS software (version 21.0; IBM Corp., Armonk, NY, USA).

## Results

### Baseline characteristics

A total of 2778 participants were included in the study and divided into two groups based on the HF type: HFmrEF/HFpEF (n = 1184) and HFrEF (n = 1594). The participants’ baseline characteristics are presented in Table 1. The average age across all participants was 67.5 years, but the HFmrEF/HFpEF group was significantly older than the HFrEF group. Sex distribution showed that males were more predominant in the HFrEF group (56.9%) than in the HFmrEF/HFpEF group (40.9%). Regarding vital signs, the HFmrEF/HFpEF group had a significantly higher average SBP than the HFrEF group, whereas the heart rate was higher in the HFrEF group. Medical history revealed that hypertension and atrial fibrillation were more common in the HFmrEF/HFpEF group, whereas a higher percentage of the HFrEF group had experienced HF, myocardial infarction, and ischemic heart disease. There were no significant differences in other conditions, including diabetes, chronic kidney disease or chronic obstructive pulmonary disease, between the two groups. Laboratory findings revealed differences in N-terminal pro B-type natriuretic peptide (NT-proBNP) levels, which were notably higher in the HFrEF group. Haemoglobin levels were significantly lower in the HFmrEF/HFpEF group than that in the HFrEF group. In terms of echocardiographic findings, the HFrEF group presented with a larger left ventricular end-diastolic diameter (LVEDD) and left ventricular end-systolic diameter (LVESD), while their LVEF was lower than that of the HFpEF/HFmrEF group. These variations underscore the distinctive clinical profiles of the HF categories.

**Table 1:** Baseline characteristics.

	All (n = 2778)	HFpEF and HFmrEF (n = 1184)	HFrEF (n = 1594)	p value
Age, year	67.5 ± 14.4	69.9 ± 13.5	65.8 ± 14.8	< 0.001
Male, n (%)	1391 (50.1)	484 (40.9)	907 (56.9)	< 0.001
BMI (> 23 kg/m^2^)	1234 (50.1)	545 (52.9)	689 (48.2)	0.022
SBP, mmHg	131.1 ± 17.7	134.7 ± 30.5	128.5 ± 28.5	< 0.001
DBP, mmHg	78.2 ± 17.7	78.2 ± 17.2	78.3 ± 18.1	0.897
Heart rate, beats/min	91.0 ± 25.0	87.7 ± 26.4	93.5 ± 23.6	< 0.001
Previous medical history, n (%)				
Heart failure	722 (28.4)	290 (26.0)	432 (30.3)	0.019
Hypertension	1297 (46.7)	620 (52.4)	677 (42.5)	< 0.001
Diabetes	844 (30.4)	357 (30.2)	487 (30.6)	0.812
Chronic kidney disease	256 (9.2)	115 (9.7)	141 (8.9)	0.438
Chronic obstructive pulmonary disease	89 (3.5)	44 (3.9)	45 (3.2)	0.279
Myocardial infarction	381 (13.7)	125 (10.6)	256 (16.1)	< 0.001
Ischemic heart disease	1029 (38.1)	410 (35.7)	619 (39.8)	0.027
Atrial fibrillation	625 (22.5)	335 (28.3)	291 (18.3)	< 0.001
Laboratory findings				
Hemoglobin, g/dL	12.4 ± 2.3	12.0 ± 2.3	12.8 ± 2.3	< 0.001
Creatinine, mg/dL	1.1 (0.9–1.5)	1.1 (0.9–1.5)	1.2 (0.9–1.5)	0.496
MDRD GFR, mL/min/1.73 m^2^	59.0 (41.8–76.8)	57.7 (40.1–78.1)	59.8 (42.9–76.5)	0.637
Serum sodium, mEq/L	139.0 (136.0–141.0)	139.0 (136.0–142.0)	139.0 (136.0–141.0)	0.822
CRP, mg/dL	2.8 ± 5.1	3.2 ± 5.5	2.6 ± 4.7	0.153
NT-proBNP, pg/mL	4508.5 (1859.0–10,579.5)	3333.0 (1220.5–8126.0)	5618.0 (2444.5–11,650.0)	< 0.001
Echocardiographic findings				
LVEDD, mm	56.0 (50.0–63.0)	51.0 (46.0–56.0)	61.0 (54.0–66.0)	< 0.001
LVESD, mm	44.0 (36.0–53.0)	35.0 (30.0–41.0)	51.0 (45.0–58.0)	< 0.001
LVEF, %	36.0 (26.0–50.0)	52.0 (45.0–61.0)	27.0 (22.0–33.0)	< 0.001

Normally distributed continuous variables are presented using mean ± standard deviation (SD), and non-normally distributed continuous variables are presented using median (25th–75th percentile).

*BMI* body mass index, *CRP* C-reactive protein, *DBP* diastolic blood pressure, *HFpEF* heart failure with preserved ejection fraction, *HFmrEF* heart failure with mildly-reduced ejection fraction, *HFrEF* heart failure with reduced ejection fraction, *MDRD GFR* modification of diet in renal disease glomerular filtration rate, *NT-proBNP* N-terminal pro B-type natriuretic peptide, *LVEDD* left ventricular end-diastolic diameter, *LVEF* left ventricular ejection fraction, *LVESD* left ventricular end-systolic diameter, *SBP* systolic blood pressure.

### Prescribing pattern at discharge according to blood pressure

Table 2 presents the prescription patterns for HF patients at discharge grouped according to SBP. The patients were categorized into two groups. In HFmrEF and HFpEF patients, a distinct pattern emerged across the five SBP ranges. As SBP increased from < 100 mmHg to ≥ 160 mmHg, the prescription rates of ARB, combinations of either ACEi or ARB, or ACEi/ARB or BB, progressively increased. The upward trend in these prescriptions was statistically significant (p < 0.001). In contrast, the prescription rates of ACEi and BB did not display a definitive trend as SBP increased. For HFrEF patients, the use of BB significantly increased as SBP levels increased (p = 0.016). In contrast, ACEi and ARB prescriptions remained relatively stable across all SBP ranges. Prescriptions of either ACEi or ARB and a combination of either ACEi/ARB or BB did not exhibit any significant trends based on SBP levels. In Supplementary Table S1, the medication prescriptions at discharge for HF patients are displayed, categorized by initial DBP levels. For HFmrEF and HFpEF patients, the data revealed an increasing trend in the prescriptions of ARB, BB, and combinations of either ACEi or ARB, or ACEi/ARB with BB. This trend became more pronounced as DBP increased from < 50 to > 110 mmHg. Notably, ACEi prescriptions did not display significant variation with changes in DBP levels. In the HFrEF group, BB prescription notably increased with increasing DBP. A similar upward trend was observed with combination therapy involving either ACEi/ARB or BB. However, the use of ACEi, ARB, or a combination of ACEi or ARB did not exhibit significant fluctuations across different DBP ranges.

**Table 2:** Initial systolic blood pressure and medication at discharge.

SBP, mmHg	< 100	100–119	120–139	140–159	≥ 160	p-for trend
HFmrEF and HFpEF patients, n (%)	(n = 97)	(n = 257)	(n = 309)	(n = 290)	(n = 231)	
ACEi	31 (32.0)	98 (38.7)	129 (42.0)	113 (39.1)	88 (38.3)	0.588
ARB	9 (9.3)	45 (17.9)	55 (18.0)	72 (25.0)	65 (28.3)	< 0.001
BB	30 (30.9)	97 (38.5)	129 (42.2)	117 (40.6)	100 (43.5)	0.064
ACEi or ARB	40 (41.2)	138 (54.5)	176 (57.3)	179 (61.9)	146 (63.5)	< 0.001
ACEi/ARB or BB	53 (54.6)	161 (63.6)	200 (65.1)	206 (71.3)	172 (74.8)	< 0.001
HFrEF patients, n (%)	(n = 174)	(n = 451)	(n = 442)	(n = 304)	(n = 223)	
ACEi	64 (37.0)	225 (50.1)	210 (47.9)	162 (53.3)	106 (47.5)	0.077
ARB	52 (30.4)	88 (19.7)	96 (22.0)	67 (22.0)	59 (26.5)	0.924
BB	58 (33.9)	183 (40.9)	195 (44.6)	134 (44.1)	103 (46.2)	0.016
ACEi or ARB	110 (63.6)	307 (68.4)	300 (68.5)	225 (74.0)	152 (68.2)	0.140
ACEi/ARB or BB	118 (68.2)	334 (74.4)	332 (75.8)	241 (79.3)	168 (75.3)	0.051

*ACEi* angiotensin-converting enzyme inhibitor, *ARB* angiotensin II receptor blocker, *BB* beta blocker, *HFmrEF* heart failure with mildly-reduced ejection fraction, *HFpEF* heart failure with preserved ejection fraction, *HFrEF* heart failure with reduced ejection fraction, *SBP* systolic blood pressure.

### Clinical outcome according to blood pressure

The relationship between the initial BP and clinical outcomes was investigated in both the HFmrEF/HFpEF and HFrEF groups (Table 3). In the HFmrEF/HFpEF group, there was no significant difference in all-cause death, or in composite events of HF readmission or all-cause death across the different SBP and DBP levels. The clinical outcomes in these patient groups did not depend significantly on the initial BP levels. In contrast, clinical outcomes in the HFrEF group showed a notable trend. With increasing SBP and DBP, there was a significant decrease in all-cause death and in composite events of HF readmission or all-cause death. This revealed a marked association between BP levels and clinical outcomes in HFrEF patients.

**Table 3:** Clinical outcomes according to initial blood pressure.

HFmrEF and HFpEF patients						
SBP level (mmHg)	< 100 (n = 97)	100–119 (n = 257)	120–139 (n = 309)	140–159 (n = 290)	≥ 160 (n = 231)	p-for trend
All-cause death, n (%)	16 (16.5)	60 (23.3)	48 (15.5)	51 (17.6)	40 (17.3)	0.336
Composite events of HF readmission or all-cause death, n (%)	32 (33.0)	1146 (45.1)	103 (33.3)	111 (38.3)	95 (41.1)	0.815
DBP level (mmHg)	< 50 (n = 31)	50–69 (n = 285)	70–89 (n = 551)	90–109 (n = 263)	≥ 110 (n = 54)	p-for trend
All-cause death, n (%)	9 (29.0)	62 (21.8)	90 (16.3)	45 (17.1)	9 (16.7)	0.063
Composite events of HF readmission or all-cause death, n (%)	12 (38.7)	119 (41.8)	207 (37.6)	99 (37.6)	20 (37.0)	0.361
HFrEF patients						
SBP level (mmHg)	< 100 (n = 174)	100–119 (n = 451)	120–139 (n = 442)	140–159 (n = 304)	≥ 160 (n = 223)	p-for trend
All-cause death, n (%)	38 (21.8)	104 (23.1)	84 (19.0)	38 (12.5)	25 (11.2)	< 0.001
Composite events of HF readmission or all-cause death, n (%)	79 (45.45)	190 (42.1)	167 (37.8)	109 (35.9)	71 (31.8)	0.001
DBP level (mmHg)	< 50 (n = 39)	50–69 (n = 409)	70–89 (n = 744)	90–109 (n = 314)	≥ 110 (n = 88)	p-for trend
All-cause death, n (%)	13 (33.3)	87 (21.3)	149 (20.0)	37 (11.8)	3 (3.4)	< 0.001
Composite events of HF readmission or all-cause death, n (%)	19 (48.7)	178 (43.5)	298 (40.1)	102 (32.5)	19 (21.6)	< 0.001

*DBP* diastolic blood pressure, *HFmrEF* heart failure with mildly-reduced ejection fraction, *HFpEF* heart failure with preserved ejection fraction, *HFrEF* heart failure with reduced ejection fraction, *SBP* systolic blood pressure.

**Figure 1:**
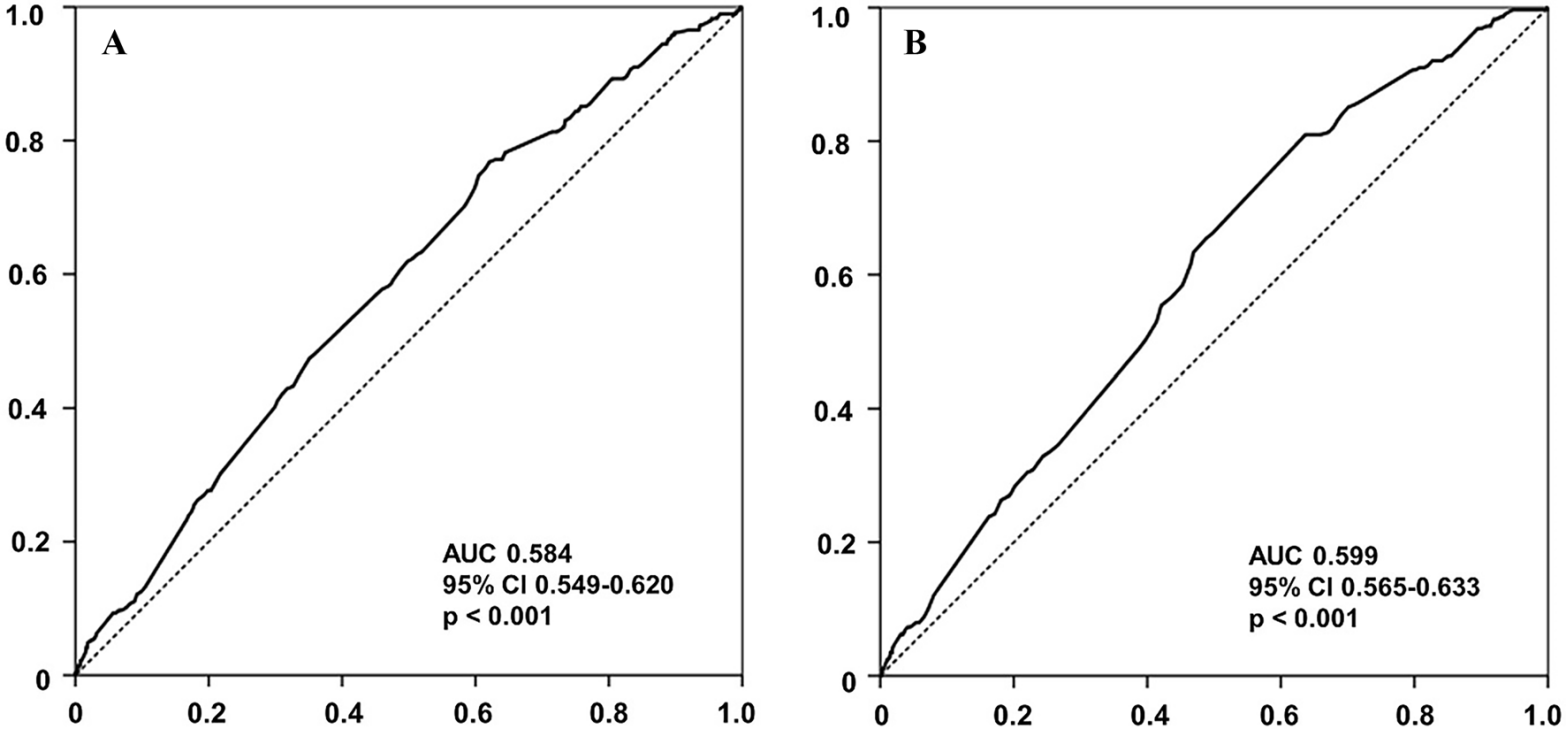
Receiver operator characteristic curve for predicting all-cause death. (**A**) Receiver operator characteristic curve for predicting all-cause death based on SBP in HFrEF patients, (**B**) Receiver operator characteristic curve for predicting all-cause death based on DBP in HFrEF patients. *AUC* area under the curve, *CI* confidence interval, *DBP* diastolic blood pressure, *HFrEF* heart failure reduced ejection fraction, *SBP* systolic blood pressure.

**Figure 2:**
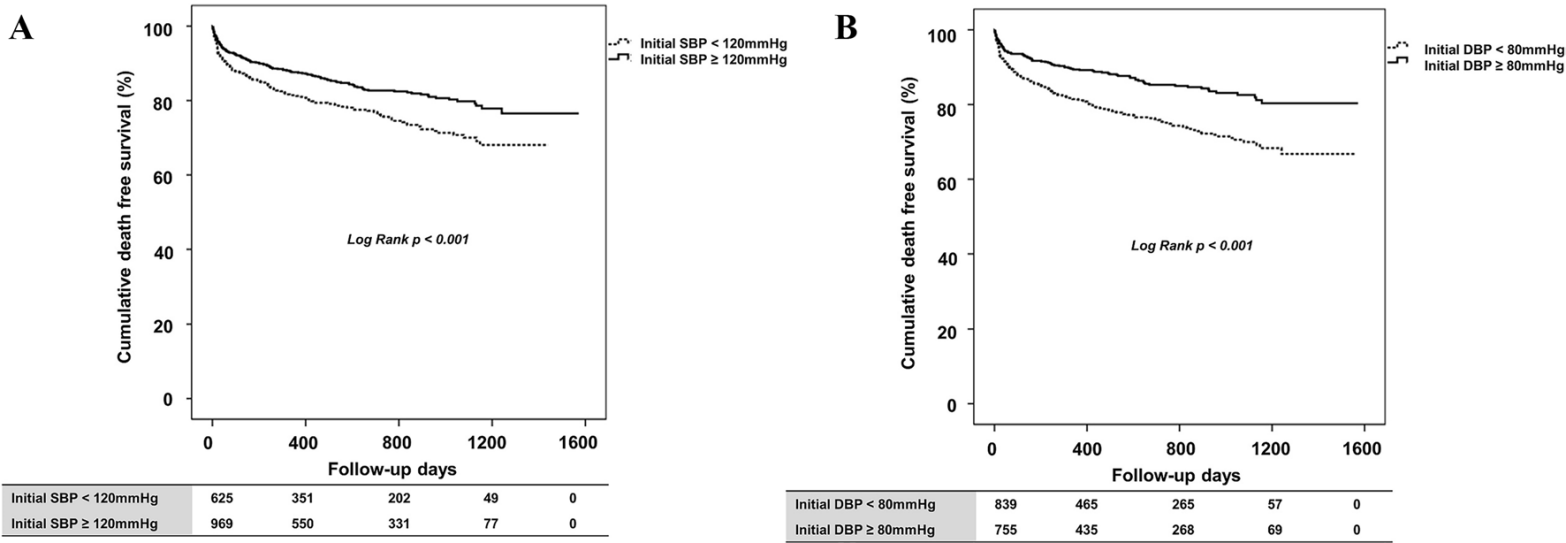
Cumulative death-free survival rate according to blood pressure. (**A**) Initial SBP < 120 mmHg and (**B**) initial DBP < 80 mmHg were associated with significantly lower death-free survival rates in HFrEF patients. *DBP* diastolic blood pressure, *HFrEF* heart failure reduced ejection fraction, *SBP* systolic blood pressure.

ROC curve analysis identified the optimal thresholds for predicting all-cause mortality based on systolic and diastolic BP values. For SBP, 120 mmHg was the best predictor of all-cause death. This threshold demonstrated a sensitivity of 57.1% and a specificity of 54.7%, as indicated in Fig. 1A. In terms of DBP (Fig. 1B), ROC curve analysis determined that 80 mmHg was the optimal cut-off value for predicting all-cause mortality. The DBP threshold exhibited a sensitivity of 66.1% and specificity of 50.3%. When stratifying the patient groups by this SBP value (Fig. 2A), those with an SBP < 120 mmHg showed significantly lower cumulative survival rates free from all-cause death than patients with an SBP ≥ 120 mmHg. The difference in survival rates was statistically significant according to the log-rank test (p < 0.001). Similar to SBP, when patients were categorized based on the DBP value, those with a DBP < 80 mmHg had a significantly lower all-cause death-free survival rate than those with a DBP ≥ 80 mmHg (Fig. 2B). Again, this discrepancy in survival rate was statistically significant according to the log-rank test (p < 0.001).

### Predictors of long-term clinical outcomes in HFrEF patients

Univariate analysis of HFrEF patients identified several significant predictors of long-term clinical outcomes, including the composite events of HF readmission or all-cause death. These included an initial SBP < 120 mmHg, an initial DBP < 80 mmHg, advanced age, presence of CKD, BMI > 23 kg/m^2^, not using ACEi or ARB, and not using BB at discharge (Table 4). After adjusting for potential confounders, except for DBP in Multivariate Model 1, an initial SBP < 120 mmHg significantly increased the risk of composite events by 1.81-fold (odds ratio [OR] 1.81, 95% confidence interval [CI] 1.349–2.417, p < 0.001). Similarly, increasing age (OR 1.03, 95% CI 1.015–1.038, p < 0.001), BMI > 23 kg/m^2^ (OR 0.72, 95% CI 0.536–0.975, p = 0.034), the presence of CKD (OR 2.04, 95% CI 1.331–3.118, p = 0.001), and the use of ACEi or ARB (OR 0.47, 95% CI 0.348–0.641, p < 0.001) and BB (OR 0.60, 95% CI 0.435–0.834, p = 0.002) at discharge remained significant. In Multivariate Model 2, an initial DBP < 80 mmHg replaced SBP and became a strong predictor of composite events (OR 2.24, 95% CI 1.645–3.053, p < 0.001). Other variables, including age, CKD, use of ACEi or ARB at discharge, and use of BB at discharge, remained significant predictors, whereas BMI > 23 kg/m^2^ showed a trend towards significance.

**Table 4:** Independent predictors of long-term clinical outcomes in patients with HFrEF.

	Univariate			Multivariate Model 1			Multivariate Model 2		
	OR	95% CI	p value	OR	95% CI	p value	OR	95% CI	p value
Initial SBP (< 120 mmHg)*	1.64	1.272–2.125	< 0.001	1.81	1.349–2.417	< 0.001	–	–	–
Initial DBP (< 80 mmHg)*	1.98	1.514–2.580	< 0.001	–	–	–	2.24	1.645–3.053	< 0.001
Age, years	1.03	1.017–1.037	< 0.001	1.03	1.015–1.038	< 0.001	1.03	1.013–1.036	< 0.001
BMI > 23 kg/m^2^	0.61	0.457–0.806	0.001	0.72	0.536–0.975	0.034	0.75	0.552–1.007	0.055
Chronic kidney disease	2.07	1.407–3.045	< 0.001	2.04	1.331–3.118	0.001	1.93	1.259–2.958	0.003
Use of ACEi or ARB at discharge	0.37	0.283–0.480	< 0.001	0.47	0.348–0.641	< 0.001	0.47	0.343–0.634	< 0.001
Use of BB at discharge	0.51	0.385–0.672	< 0.001	0.60	0.435–0.834	0.002	0.58	0.422–0.810	0.001

*Cut-off value of the ROC curve.

*ACEi* angiotensin-converting enzyme inhibitor, *ARB* angiotensin II receptor blockers, *BB* beta-blockers, *BMI* body mass index, *DBP* diastolic blood pressure, *HFrEF* heart failure reduced ejection fraction, *OR* odds ratio, *SBP* systolic blood pressure.

## Discussion

This comprehensive study investigated the effect of BP admission on long-term clinical outcomes in patients with acute HF before the era of new drugs (angiotensin receptor neprilysin inhibitors and sodium-glucose cotransporter-2 inhibitors). Distinct acute HF patient groups were identified: HFrEF and HFmrEF/HFpEF. Notably, only the HFrEF group showed a significant inverse relationship between the initial BP and outcomes, including all-cause death and HF readmission. However, this relationship was not observed in HFmrEF/HFpEF patients. Furthermore, HFrEF patients admitted with a systolic BP < 120 mmHg or diastolic BP < 80 mmHg had significantly more adverse clinical outcomes. Other factors, such as older age, presence of CKD, higher BMI, and the absence of ACEI or ARB, and BB upon discharge, were also notable predictors of worse clinical outcomes in the HFrEF patient group. These findings shed light on the complex relationship between initial BP and long-term clinical outcomes in acute HF patients. These findings could be pivotal in refining treatment strategies to improve the prognosis of HFrEF patients.

Consistent with our findings, international guidelines have emphasize the importance of BP management in HF patients^12–14^. The European Society of Cardiology (ESC) 2023 guidelines highlight the need to monitor and manage BP in HF patients to optimize treatment outcomes^12^. Specifically, they recommend that for general HF prevention in hypertensive patients, SBP should be reduced to < 140 mmHg, with evidence suggesting benefits in the 129–120 mmHg range for HF. For patients with HFrEF, an SBP < 130/80 mmHg is suggested, whereas in HFpEF, an SBP of 130 mmHg is recommended. Similarly, the American College of Cardiology and American Heart Association recognize BP control as crucial for HF management^13^. They advise a target BP < 130/80 mmHg for general hypertension linked to HF risk, referencing the SPRINT trial findings^15^. HFrEF patients with hypertension are advised to maintain a BP < 130/80 mmHg using guideline-directed medical therapy. For HFpEF, managing hypertension with diuretics is suggested, especially in the presence of volume overload symptoms. ACEi, ARB, and BB should be considered if hypertension remains uncontrolled and with the aim of achieving an SBP < 130 mmHg. The Korean Society of Cardiology concurs with these guidelines, underscoring the risks of uncontrolled BP extremes in HF patients and emphasizing regular monitoring and interventions^14^. They advise a target BP < 130/80 mmHg for HF patients. Therefore, global cardiological societies have consistently highlighted the pivotal role of BP in the management and treatment of HF patients, emphasizing its clinical importance.

The pivotal role of BP regulation in the complex field of HF management is becoming increasingly evident. For those hospitalized with acute HF exacerbation, the initial BP provides valuable insight into the effectiveness of BP management. Examination of the HF subtypes further reveals nuances. Surprisingly, in HFrEF patients, a higher initial BP indicated a decreased risk of adverse events, including all-cause death and HF readmission. This aligns with the concept of the ‘blood pressure paradox’ wherein a traditionally high BP, often considered detrimental, paradoxically confers survival advantages in the context of chronic HF^16–19^. This counterintuitive phenomenon has garnered attention in HF studies, including the influential works by Schmid et al.^17^ Gheorghiade et al.^18^, and Lee et al.^19^ underlining the necessity for a refined understanding of the clinical management of these patients.

An important aspect of our study was the identification of ideal BP thresholds for predicting poor clinical outcomes in HFrEF patients. We pinpointed 120 mmHg and 80 mmHg as the optimal systolic and diastolic BP thresholds, respectively, for predicting all-cause mortality. Notably, patients with an initial BP below these thresholds exhibited significantly lower survival rates. These insights have substantial clinical implications for risk stratification and therapeutic decision making in HFrEF patients. Further supporting our findings, a previous study by Gheorghiade et al.^18^ found that a lower admission BP was a predictor of mortality in HF patients, emphasizing the connection between BP and long-term survival in HF patients. In addition, a significant study by Böhm et al.^20^ noted that HF patients with lower SBP experienced worse outcomes, specifically an increased rate of primary composite outcomes, including cardiovascular death, HF hospitalization, and all-cause death.

Our insights highlight the nuanced relationship between initial BP and discharge prescription patterns in patients with HF, which are influenced by specific clinical considerations. It is evident from our data that SBP influences discharge prescriptions in a manner dependent on the HF subtype. The increasing prescription rates with higher SBP in the HFmrEF and HFpEF groups may reflect clinical concerns related to potential hypertensive damage or worsening heart function in these subgroups^2,21^. In the HFrEF group, the rising preference for BB with increased SBP could be driven by their proven efficacy in attenuating the detrimental effects of the heightened sympathetic activation commonly seen in this cohort^22,23^. Similarly, DBP variations at discharge significantly influenced the medication choices. In the HFmrEF and HFpEF groups, increasing DBP levels correlated with augmented ARB, BB, and combinations of either ACEi or ARB, or ACEi/ARB with BB prescriptions. This might be due to the recognized role of these medications in mitigating diastolic dysfunction or addressing the underlying comorbid conditions in these subtypes^24,25^. The increased preference for BB in the HFrEF group with elevated DBP further cements their role in modulating afterload and addressing elevated cardiac stress, consistent with SBP-driven observations^10,26^.

This study offers a comprehensive exploration of relationship between initial BP and clinical outcomes in acute HF patients, particularly highlighting new insights into the ‘BP paradox’ for HF. It is crucial to consider the historical context of our data, collected before the introduction of angiotensin receptor neprilysin inhibitors and sodium-glucose cotransporter-2 inhibitors, now standard in HF treatment. As these therapies have markedly improved outcomes, our study’s applicability to current practice may be limited, highlighting the need for ongoing research to update blood pressure management strategies in light of these advancements.

Several limitations may have affected our findings. First, our analysis was based on a single-point measurement of SBP and DBP, which may not accurately reflect patients’ long-term BP control status. BP can fluctuate significantly throughout the day and can be influenced by various factors such as physical activity, emotional state, and medication use. Multiple readings over time can provide more accurate assessment. Second, this study was observational in nature. Although we employed rigorous statistical methods to control confounding variables, there may have been residual confounders that were not considered. Third, our study's inclusion of both patients with de novo HF and those presenting with acute decompensation of chronic HF introduces significant heterogeneity that could complicate the interpretation of our findings. While representative of a real-world clinical scenario, this diverse patient composition means that treatment responses and clinical outcomes may vary widely. Chronic HF patients often receive ongoing, evidence-based treatments that could influence their prognosis differently compared to those newly diagnosed. Recognizing these differences is crucial for accurately assessing the impact of initial BP and other factors on long-term outcomes. Additionally, our study's timeframe (2004–2009) predates the widespread implementation of current HF treatment guidelines recommending more aggressive use of BB in HFrEF. As such, the BB prescription rates reported in our study reflect earlier practices, which may not correspond to current treatment outcomes or standards. Fourth, the diagnosis of HF was based on the Framingham criteria, which might be less effective in capturing cases of HFpEF, particularly in the presence of overlapping symptoms from conditions such as chronic obstructive pulmonary disease. This diagnostic approach might limit the accuracy in identifying the full spectrum of HF presentations, particularly among those with complex comorbidities. Furthermore, the absence of detailed follow-up information on medication titration in our study is a significant limitation. The lack of data on attempts to up-titrate HF medications to target doses means our findings regarding initial BP values must be interpreted cautiously. Additionally, the inclusion of a significant proportion of post-myocardial infarction patients without comprehensive medication tracking and classification could compromise the interpretation and generalizability of our results. It is well-established that achieving optimal medication doses is crucial for improving outcomes in chronic HF, and our study does not address how changes in treatment regimens might have impacted the observed outcomes. Lastly, while we identified specific cut-off points for SBP and DBP that were associated with all-cause mortality, these thresholds may not apply equally to all patient subgroups. Further research is needed to investigate the applicability of these cut-off values across different demographic groups and those with varying comorbid conditions. Despite these limitations, our study provides valuable insights into the potential relationship between BP and clinical outcomes and can guide future research in this field.

In conclusion, this study revealed a significant inverse association between the initial BP and long-term clinical outcomes in patients with HFrEF. Furthermore, adverse clinical outcomes were more likely in HFrEF patients admitted with an SBP < 120 mmHg or DBP < 80 mmHg. These findings emphasize the complexity of the relationship between initial BP and long-term outcomes in acute HF patients and could contribute to the refinement of treatment strategies aimed at improving prognosis in HFrEF patients.

### Supplementary Information

Supplementary Table S1.

## Data Availability

The datasets generated during the current study are available from the corresponding author on reasonable request.
