# Supplementary material for: Effect of low blood pressure on prognosis of acute heart failure
Source: Sci Rep. 2024 Jul 6;14:15605. doi: 10.1038/s41598-024-66219-2 (PMC11227539; doi:10.1038/s41598-024-66219-2)
Supplement: Supplementary file 1 — Supplementary Table S1. [file 41598_2024_66219_MOESM1_ESM.pdf]

Supplementary Material

Supplementary Table S1. Initial diastolic blood pressure level and medication at discharge

DBP level (mmHg)	<50	50-69	70-89	90-109	≥110	p-for trend
HFmrEF and HFpEF patients, n (%)	(n = 31)	(n = 285)	(n = 551)	(n = 263)	(n = 54)	
ACEi	9 (29.0)	113 (39.9)	215 (39.3)	99 (37.9)	23 (42.6)	0.749
ARB	2 (6.5)	50 (17.7)	110 (20.1)	71 (27.3)	13 (24.1)	0.002
BB	9 (29.0)	107 (37.9)	212 (38.8)	119 (45.8)	26 (48.1)	0.013
ACEi or ARB	11 (35.5)	157 (55.5)	314 (57.4)	162 (62.1)	35 (64.8)	0.040
ACEi/ARB or BB	17 (54.8)	180 (63.6)	363 (66.4)	194 (74.3)	38 (70.4)	0.004
HFrEF patients, n (%)	(n = 39)	(n = 409)	(n = 744)	(n = 314)	(n = 88)	
ACEi	14 (36.8)	187 (45.7)	372 (50.3)	154 (49.0)	40 (46.0)	0.315
ARB	8 (21.6)	90 (22.2)	156 (21.1)	80 (25.5)	28 (32.2)	0.055
BB	9 (24.3)	159 (39.2)	320 (43.4)	138 (43.9)	47 (54.0)	0.003
ACEi or ARB	21 (55.3)	271 (66.3)	518 (70.1)	223 (71.0)	61 (70.1)	0.064
ACEi/ARB or BB	22 (57.9)	295 (72.1)	567 (76.7)	239 (76.1)	70 (80.5)	0.013

ACEi, Angiotensin-Converting Enzyme inhibitors; ARB, Angiotensin II Receptor Blockers; BB, Beta Blockers; DBP, diastolic blood pressure; HFmrEF, Heart Failure with mildly-reduced Ejection Fraction; HFpEF, Heart Failure with preserved Ejection Fraction; HFrEF, Heart Failure with reduced Ejection Fraction.
